# True Colour Classification of Natural Waters with Medium-Spectral Resolution Satellites: SeaWiFS, MODIS, MERIS and OLCI

**DOI:** 10.3390/s151025663

**Published:** 2015-10-09

**Authors:** Hendrik J. van der Woerd, Marcel R. Wernand

**Affiliations:** 1 Royal Netherlands Institute for Sea Research, Physical Oceanography, Marine Optics & Remote Sensing, PO box 59, Den Burg1790AB, Texel, The Netherlands; 2 Institute for Environmental Studies (IVM), VU University Amsterdam, De Boelelaan 1087, Amsterdam 1081HV, The Netherlands

**Keywords:** ocean colour remote sensing, spectral bands, hue angle, citizen science, MERIS, MODIS, SeaWiFS, OLCI, colourimetry, water quality

## Abstract

The colours from natural waters differ markedly over the globe, depending on the water composition and illumination conditions. The space-borne “ocean colour” instruments are operational instruments designed to retrieve important water-quality indicators, based on the measurement of water leaving radiance in a limited number (5 to 10) of narrow (≈10 nm) bands. Surprisingly, the analysis of the satellite data has not yet paid attention to colour as an integral optical property that can also be retrieved from multispectral satellite data. In this paper we re-introduce colour as a valuable parameter that can be expressed mainly by the hue angle (α). Based on a set of 500 synthetic spectra covering a broad range of natural waters a simple algorithm is developed to derive the hue angle from SeaWiFS, MODIS, MERIS and OLCI data. The algorithm consists of a weighted linear sum of the remote sensing reflectance in all visual bands plus a correction term for the specific band-setting of each instrument. The algorithm is validated by a set of 603 hyperspectral measurements from inland-, coastal- and near-ocean waters. We conclude that the hue angle is a simple objective parameter of natural waters that can be retrieved uniformly for all space-borne ocean colour instruments.

## 1. Introduction

Dedicated satellite observations of the oceans were introduced with the launch of the Coastal Zone Colour Scanner (CZCS) in 1978 [1]. Since then sensors for worldwide observations of natural waters, notably the Sea-viewing Wide Field-of-view Sensor (SeaWiFS), the Moderate Resolution Imaging Spectroradiometer (MODIS) and Medium Resolution Imaging Spectrometer (MERIS), have been in space with a relatively short interruption (June 1986 to September 1997). One of the next generation imaging spectrometers is the Ocean and Land Colour Instrument (OLCI) on the Sentinel-3 platform. These instruments have been developed towards spectrometers that select only a limited number of narrow (≈10 nm) spectral bands, mostly in the visual domain, to feed algorithms for the detection of the water composition, like algal pigments, notably chlorophyll-a, suspended particulate matter and Coloured Dissolved Organic Matter [2,3].

The increased sensitivity over time of the sensors and the addition of narrow bands in the near-infrared (700–800 nm) made it possible to derive a much better atmospheric correction over coastal and inland waters with increased spatial resolution (300 m). Satellite data have tremendously stimulated efforts to develop new algorithms for the retrieval of water constituents [4]. Many of these algorithms focus on the use of only a limited number of sensor bands, although a few tried to reconstruct the full spectrum [5].

Colour is a concept that originates in the human perception of radiation between the (extreme) values of 380 to 720 nm. The human eye has three cone receptors that are very sensitive in the red, green or blue. Since the start of the 20th century the sensitivity of human colour perception was well documented [6]. Also scientists like Forel and Ule found a way for a consistent measure of “the water colour” by using human perception to compare colours of natural waters. This Forel-Ule (FU) scale is a historical standard that has recently been very well calibrated [7]. The scale was developed because of technological limitations that existed at the end of the 19th century. However, new initiatives in participatory science like within the EU-Citclops project [8], indicate that the colour-comparison methodology can be transferred to nowadays measuring techniques using smart phones and other new kind of devices [9].

In aquatic optics there is a fundamental difference between “apparent” and “inherent” optical properties of water. Apparent properties are influenced by environmental conditions, for example the total radiation coming from the water surface, because they are influenced by sky reflection and the illumination spectrum, determined by solar and sky radiation. Inherent properties are independent of environmental conditions and can be used to classify natural waters, for example the absorption spectrum (m^−1^), scattering spectrum (m^−1^) and volume scattering function (dimensionless). If we follow this general idea, it is clear that the spectrum of remote sensing reflectance (*R*_RS_ in sr^−1^ units) is an apparent optical property. However, as long as the standard satellite atmospheric corrections provide a uniform *R*_RS_ product, quasi-independent of illumination conditions, the colour of an infinite deep open water depends only on the composition of the water [10,11]. As the spectral distribution is difficult to classify, we propose to use the standard 1931 CIE [6] Colour Matching Functions (CMF) to convert the light spectrum into three values; the *x*, *y*, *z* chromaticity coordinates. Those values can be further compressed into one value: The hue angle (α) that will be referred to as the true colour of natural waters.

Current satellite ocean colour imaging instruments are multi-spectral, meaning they detect only part of the electromagnetic spectrum; therefore, a straight-forward calculation of the true colour is not possible, like in the case of hyperspectral data. However, the colour of natural waters can be reconstructed well based on a limited set of spectral bands, since R_RS_ spectra are not an arbitrary function with extreme variations in the wavelength dependence (see Lee *et al.* [3]). It is true that the concentration of the optically active substances in the water can vary substantially, but the effect on the true colour is normally characterized by smooth transitions. In a first study, Wernand *et al.* [12] demonstrated that the MERIS band setting allows for very accurate reconstruction of the water colour. After the development of the FUME algorithm [12], all MERIS FR data near the Spanish Mediterranean coast have been processed to hue angle and Forel-Ule scale for the Citclops project [8]. From a first inspection of these maps, a number of applications have become apparent—easy descriptor of (rapidly changing) events like river plumes, eddies, convergence of water masses—quick check for (small) observation errors like atmospheric correction near clouds and close to land—connection to the water-observation experience of citizens. Also it became apparent that, although the 21 Forel-Ule numbers connect historical data to present observations, the hue angle is a more precise quantification (per degree over the range 20° to 230°).

In this paper we generalize this idea to more satellite instruments and present a simple algorithm to derive the true water colour for past, present and future ocean colour imaging spectrometers. The algorithm is based on a set of 500 synthetic *R*_RS_ spectra [13] plus 603 *R*_RS_ field spectra with relatively high spectral resolution (10 nm and 3.3 nm, respectively). For each spectrum the chromaticity coordinates and hue angle (α) can be calculated with high precision. Subsequently, the *R*_RS_ at only the satellite bands is extracted from the full spectra and the satellite hue angle is calculated based on this information alone. We find differences between the two calculated hue angles, but these difference follow a well-defined curve that allows an empirical correction.

## 2. Material and Methods

### 2.1. Satellite Colourimetry

The derivation of the true colour of natural waters is based on the calculation of the Tristimulus values that are the three primaries (*X*, *Y*, *Z*) that specify a colour stimulus of the human eye [6,14]. Suppose the radiation spectrum that comes from the water is given by *I* that is a function of wavelength (λ), then the tristimulus values are given by:

(1a) $$X=\int I\left(\lambda \right)\bar{x}(\lambda )d\lambda$$

(1b) $$Y=\int I\left(\lambda \right)\bar{y}(\lambda )d\lambda$$

(1c) $$Z=\int I\left(\lambda \right)\bar{z}(\lambda )d\lambda$$

The CIE 1931 standard colourimetric two degree Colour Matching Functions (CMFs) are presented by *x* (red), *y* (green) and *z* (blue). These serve as weighting functions for the determination of the tristimulus values. The intensity *I* can be replaced by *I* = *E* × *R*, the product of the illumination *E* times the remote sensing reflectance of water (*R*) [10]. For notation purposes we introduce the symbol *T* that represent the three tristimulus values (*X*, *Y*, *Z*) and $(\bar{t)\;}$ that represents the three CMFs:

(2) $$T=\int E(\lambda )R_{\text{RS}}\left(\lambda \right)\bar{t}(\lambda )d\lambda$$

To further simplify the calculations the illumination *E* is taken as a constant, independent of wavelength, and the remote sensing reflectance is assumed to be corrected for the surface effects (Fresnel reflectance, foam, capillary waves). We refer to the standard books on water remote sensing by Mobley [11] and Kirk [10]. Thus *R*_RS_ (λ) describes the intrinsic colour of the water, independent of air-water interface effects or illumination effects. Finally, because the integrals cannot be solved analytically, *T* can be written as the summation:

(3) $$T=E\sum_{i=400}^{710}R_{\text{RS}}(\lambda )\bar{t}(\lambda )\Delta \lambda \text{ or }T=\sum_{i=400}^{710}y(\lambda )\Delta \lambda \text{ or }T=\sum_{i=400}^{710}\Delta T$$

Note that the summation is taken between 400 and 710 nm. This will be discussed in more detail below. Also *E* is taken as unity and y is the product of the remote sensing reflectance times the CMF weighting functions:

(4) $$y(\lambda )=R_{\text{RS}}(\lambda )\bar{t(\lambda )}$$

**Figure 1:**
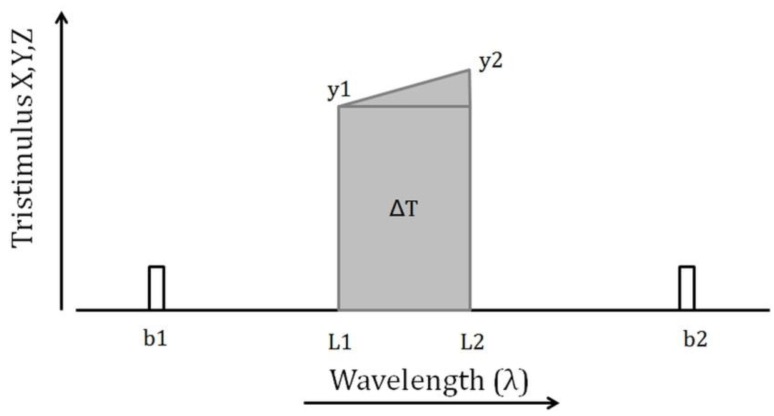
Diagram to show the contribution of a small part of the spectrum, lying between bands b1 and b2, to the tristimulus values (ΔT).

Because ocean colour satellites do not provide full-spectral coverage, the *y*-spectrum must be first reconstructed by linear interpolation, based on the remote sensing reflection measured at the spectral bands (b). The contribution to *T* of a small interval of the spectrum between wavelengths L1 and L2 can be approximated by the trapezium rule (Figure 1):

Δ*T* = (*L2* − *L1*) (*y1* + *0.5* (*y2* − *y1*)) = *0.5* (*L2* − *L1*) (*y1* + *y2*) (5)

To calculate y (Equation (4)), *R*(λ) = *R*_RS_(λ) must be retrieved from the values at the satellite bands b1 and b2. This can be done by linear interpolation at wavelength L1 and L2:

*R*_(L1,L2)_ = *R*_b1_ + (*R*_b2_ − *R*_b1_) (L_1,2_ − b1)/(b2 − b1) (6)

If Equations (4)–(6) are combined we find:

Δ*T* = A (t_L1_(*R*_b1_ + B (*R*_b2_ − *R*_b1_)) + t_L2_(*R*_b1_ + C (*R*_b2_ − *R*_b1_))) (7)

with:

*A* = *0.5* (*L2* − *L1*); *B* = (*L1* − *b1*)/(*b2* − *b1*); *C* = (*L2* − *b1*)/(*b2* − *b1*) (8)

Rewriting Equation (7) to an expression that is linear in the satellite bands *R*_b1_ and *R*_b2_ we find:

Δ*T* = *R*_b1_ A (t_L1_ (1 − B)+t_L2_ (1 − C)) + *R*_b2_ A (t_L1_ (B) + t_L2_ (C)) (9)

This implies that if we have the measured R values at b1 and b2, we can estimate ΔT between those bands (Equations (3), (8) and (9)) as a linear combination of those two, because for every wavelength interval we can calculate A, B and C and know $(\bar{t)}$ at wavelengths L1 and L2 from [6]. Once the tristimulus values *T* (*X*, *Y*, *Z*) have been calculated, the three values are normalized and the colour is expressed in the coordinates:

(10) $$x=\frac{X}{X+Y+Z}\;y=\frac{Y}{X+Y+Z}$$

**Figure 2:**
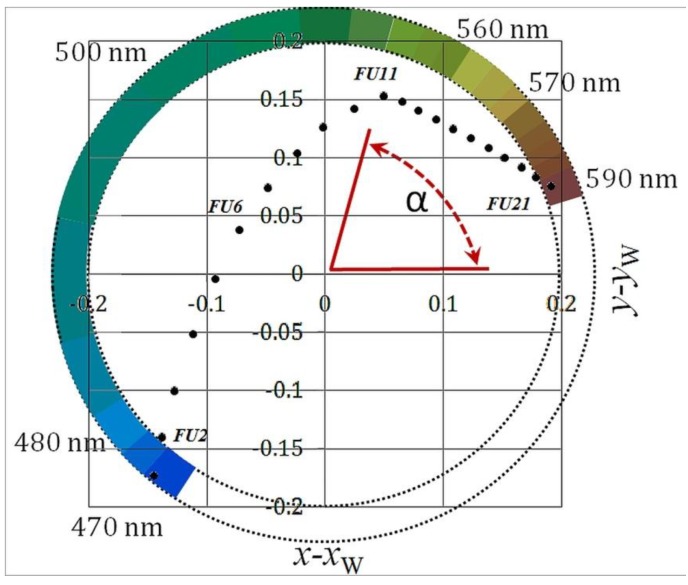
A chromaticity diagram showing the hue colour angle (α) match relative to the white point (*x*_w_, *y*_w_) of the FU scale colours. The dominant wavelength of the specific segment is indicated in nm.

The white point has the coordinates *x* = *y* = 1/3. In the (*x*, *y*) chromaticity plane, the coordinates are transformed to polar coordinates with respect to the white point and the hue angle is derived. The hue angle (α) lies between the vector to a point with coordinates (*x* − *x_w_*, *y* − *y_w_*) and the positive x-axis (at *y* − *y_w_* = 0), giving higher angles in an anti-clockwise direction (see Figure 2).

(11) $$\alpha =\arctan\left(y-y_{W},x-x_{W}\right)\text{ modulus 2}\pi$$

All calculations in this paper were made with the ATAN2 function (four-quadrant inverse tangent) and the derived angles (in radians) are multiplied by 180/π to get the angles in degrees. In the development of the water hue angle concept, Wernand *et al.* [12] used (α*_M_*) for the hue angle derived with the FUME algorithm for MERIS, while Novoa *et al.* [15] introduced (α*_W_*) for the hue angle of water. In this manuscript we will refrain from indices and use (α) as the hue angle that represents the “true” or “intrinsic” colour of a natural water, which can be approximated by satellite remote sensing reflectance measurements.

### 2.2. Data

#### 2.2.1. Synthetic Spectra

**Figure 3:**
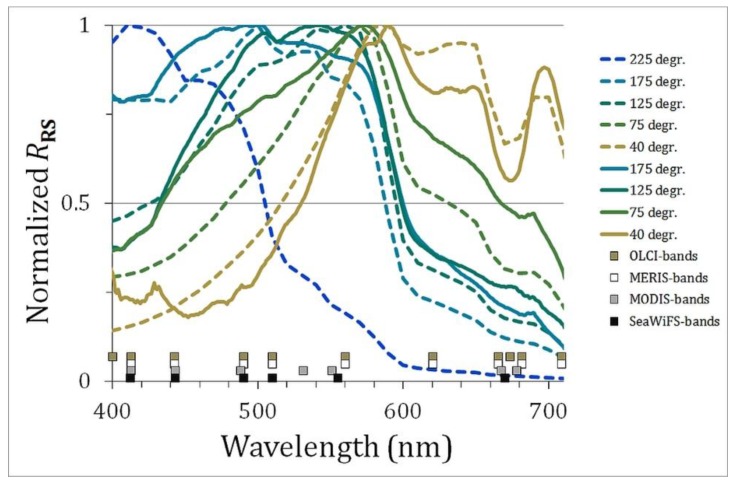
Example of synthetic (full lines) and field (dashed lines) spectra with hue angles varying between 225° and 40°. Spectral satellite bands are given for OLCI, MERIS, MODIS and SeaWiFS.

In order to develop and test the algorithms, synthetic Remote Sensing reflectance data (*R*_RS_) data from [2] were used to define a set of spectra that can be expected from natural waters. The above water Rrs was simulated using Hydrolight [16] and a set of inherent optical properties derived from extensive field measurements. Details on this data set can be found in [2] and the IOCCG website [13]. This synthetic data is generally used as a good benchmark for algorithm development [17], because it covers a wide range in natural-water composition without errors from measurement procedures. The 500 synthetic *R*_RS_ spectra are provided every 10 nm between 400 and 800 nm. Examples of the typical shape of these spectra are shown in Figure 3, together with the positions of wavelength bands from four multi-spectral imagers.

The true colour was derived by interpolation of the IOCCG data to a 1 nm grid and calculation of (α) by Equations (3), (4), (10) and (11). Each spectrum was converted to the multiband spectra for the instruments MERIS, OLCI, MODIS and SeaWiFS by linear interpolation of the synthetic *R*_RS_ values at the 10 nm grid. The properties of these instruments are summarized in Table 1. For each band the band number, central wavelength and band width in nm are given. The last bands given in this table are outside the visual domain and are mainly used for atmospheric correction procedures, with the exception of extremely turbid waters [18].

**Table 1:** Band settings of ocean colour instruments. The central wavelength and band width are given in nm. Bands in bold with underscore are outside visual domain.

Instrument	SeaWiFS	MODIS Aqua	MERIS	Sentinel 3 OLCI
**Operational**	September 1997–December 2010	May 2002–present	May 2002–April 2012	End 2015–
**Spatial Res.**	1100 m	1000 m	300 m	300 m
**Band nr.**	Wavelength/Bandwidth			
**1**	412/20		412.5/10	400/15
**2**	443/20		442.5/10	412.5/10
**3**	490/20		490/10	442.5/10
**4**	510/20		510/10	490/10
**5**	555/20		560/10	510/10
**6**	670/20		620/10	560/10
**7**	**765/40**		665/10	620/10
**8**		412.5/15	681.25/7.5	665/10
**9**		443/10	708.75/10	673.5/7.5
**10**		488/10	**753.75/7.5**	681.25/7.5
**11**		531/10		708.75/10
**12**		551/10		**753.75/7.5**
**13**		667/10		
**14**		678/10		
**15**		**748/10**		

This brings us to the wavelength intervals that have to be considered in this study. The tristimulus response curves of the human eye quickly drop to very small values below 400 nm and above 700 nm, implying that these regions contribute marginally to the hue angle. Because the smallest wavelength of the IOCCG data set is 400 nm and the OLCI instrument on the Sentinel-3 mission will have a band centred at 400 nm, this wavelength is used as the lower boundary of our algorithms. Because the absorption by pure water increases exponentially above 700 nm and CDOM and algae absorb marginally above 700 nm, it makes sense to set the upper wavelength limit at 700 nm. However, for sediment rich waters there is still some reflection above 700 nm and the MERIS band 9 at 708 nm has proven to be very valuable for studies of so-called case-2 waters [19]. As a compromise we take the cut off at 710 nm and the hue angle discussed in this paper is based on the interval 400–710 nm.

#### 2.2.2. TriOS Spectra

Validation of the algorithms is carried out by analysis of hyperspectral *R*_RS_ *in situ* measurements that were collected for the EU-Citclops project [8], in order to validate innovative and dedicated smartphone applications [15]. A total of 603 spectra were collected in 2013 at 43 sampling stations in the North Sea and Dutch coastal and inland water bodies. More information can be found in [15]. At each station hyperspectral measurements were carried out using TriOS-RAMSES radiometers following the NASA protocols [20]. The measurements included sky radiance (*L_sky_*), upwelling radiance (*L_sfc_*) and incident spectral irradiance (*E_S_*). The radiometers cover the spectral range 320–950 nm with a spectral resolution of 3.3 nm (Full Width at Half Maximum) and an accuracy of 0.3 nm. Radiance measurements were collected at an azimuth angle of 135° away from the Sun. Sky and water surface radiance were measured at 35° off zenith and nadir respectively. The water-leaving radiance L_W_ (λ, 0^+^) at wavelength (λ) just above the surface (0^+^) is derived from the following:

*L_W_* (λ, 0^+^) = *L_sfc_* (λ, 0^+^) − ρ·*L_sky_*(λ) (12)

The reflectance factor ρ is a correction factor to compensate for Fresnel reflectance at the air/water boundary [20], defined as the fraction of skylight actually reflected from the wave roughened (sea) surface. In this study we simply calculated ρ by demanding that water-leaving radiance at 360 nm equals zero. The intrinsic colour of the water is given by the spectral distribution of the remote-sensing reflectance *R*_RS_ (λ, 0^+^) that is calculated as the ratio of water-leaving radiance *L*_W_ (λ, 0^+^) over down-welling irradiance *E*_S_ (λ, 0^+^):

*R*_RS_= *L_W_* (λ, 0^+^)/*E_S_* (λ, 0^+^) (13)

Examples of the real spectra are also plotted in Figure 3, next to synthetic data of the same hue angle and the band setting of the satellite instruments. Again the intrinsic colour (true colour) was calculated by interpolation of the TriOS data to a 1 nm grid and calculation of (α) by Equations (3), (4), (10) and (11). Each spectrum was converted to a multiband spectrum for the specific satellite instruments by linear interpolation.

#### 2.2.3. Satellite Data

For inter-comparison of the multi-spectral satellite images, MERIS, MODIS-Aqua and SeaWiFS images were processed to hue angle. From the NASA website for ocean colour observations (oceancolor.gsfc.nasa.gov), the level-2 products MER_RR_2PQBCM20060504_101124_xx, S2006124121824SW and a2006124115500.l2_LAC_OC were retrieved. These images cover the North Sea area, notable the central North Sea and the coastal water of The Netherlands, Germany and Denmark. The images were collected at 4 May 2006 (UTC 10:14, 11:55 and 12:18 hours respectively). First the satellite images were re-projected into Lambert Azimuthal Equal area by means of VISAT-Beam software [21]. Each image was converted through Equations (3)–(11), with the instrument-specific coefficients to respectively the tristimulus value X, Y, Z, the chromaticity coordinates x, y and the hue angles α. Through the VISAT-Beam collocation function, the MODIS and SeaWiFS image were transferred by nearest-neighbour interpolation to the same grid (same pixel size) as the MERIS image.

## 3. Results

### 3.1. Simulation Spectra

The hue angles for the 500 synthetic spectra were determined and the satellite-based hue angle was calculated for the instruments MERIS, MODIS, SeaWiFS and OLCI. First we concentrate on the MERIS instrument and explain in more detail the outcome, before the results of all four ocean colour instruments are presented. In Table 2 the result of Equation (9) is presented for the nine MERIS bands. The summation between the bands is carried out in steps of 1 nm. This table can be used as follows: Once the MERIS *R*_RS_ is known on all nine bands, the tristimulus values can be calculated as a linear sum of the band information. For example the blue value *Z*:

(14) $$Z=\sum_{i=1}^{i=9}ZM(i)R_{\text{RS}}(i)$$

In case independent information exists about the *R*_RS_ at 400 nm or 710 nm; for example by regional knowledge of the characteristic reflection spectra; two extra terms can be added: *Z* = *Z* + 0.731 * *R*_RS400_ + 0.000 * *R*_RS710_. For a pure white reflection spectrum R can be taken out of the summation in Equation (14) and we find the sum of the coefficients (*X*, *Y*, *Z*) = (106.665; 106.824; 106.335) and the resulting (*x*, *y*) coordinates (Equation (10)) are (0.3335; 0.3340); very close to the theoretical white point at (1/3; 1/3). From Table 2 we can see that the dominant contribution for red; green and blue comes from bands 6; 5 and 2, respectively.

**Table 2:** Linear coefficients to calculate the chromaticity values based on MERIS bands.

ME-Band		1	2	3	4	5	6	7	8	9	
λ (nm)	400	412.5	442.5	490	510	560	620	665	681.25	708	710
x_ME_	0.154	2.813	10.867	3.883	3.750	34.687	41.853	7.619	0.844	0.189	0.006
y_ME_	0.004	0.104	1.687	5.703	23.263	48.791	23.949	2.944	0.307	0.068	0.002
z_ME_	0.731	13.638	58.288	29.011	4.022	0.618	0.026	0.000	0.000	0.000	0.000

**Figure 4:**
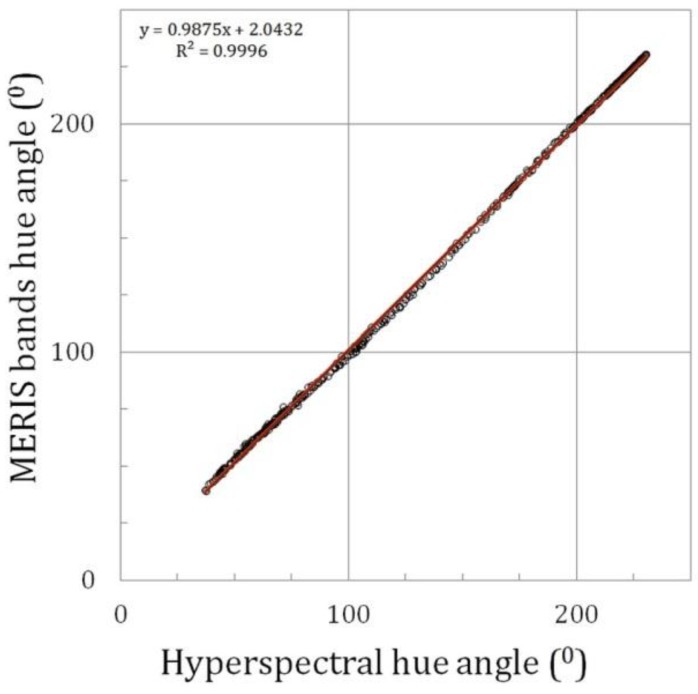
The true hue angle as function of the linear hue angle derived from the MERIS band combination.

A final test the linear band approximation (Equation (14)) was made by comparing the hue angle of the synthetic data with the values derived from the original summation of y values (Equation (3)): The resulting correlation is almost perfect with small rounding off errors with standard deviation of 0.22°. This shows that Equation (14) can be used for a simple calculation of the hue angle from medium resolution spectra. But how well can the data selection on MERIS wavebands, that cover only a limited view of underlying reflection spectrum, be used to reconstruct the true colour? The answer is presented in Figure 4. This figure confirms that the synthetic data set smoothly covers a large range in hue angles, ranging between 231° (indigo blue oligotrophic oceanic waters) and 37° (red-brown CDOM rich waters). A linear relation works very well with only a small standard deviation of 1.6°. MERIS band setting is therefore very appropriate for retrieving the intrinsic colour of natural waters and it confirms the findings of an earlier publication on the FUME algorithm [12].

**Figure 5:**
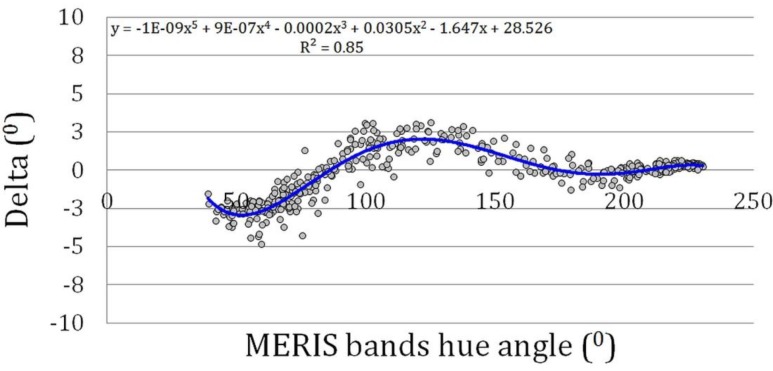
Deviation delta (°) from the hyperspectral hue angle as function of the MERIS hue angle derived from the linear satellite band combination.

However, if we study the relation in more detail, a systematic deviation delta (Δ) from the calculated hyperspectral hue angle as function of the MERIS calculated hue angle can be detected, see Figure 5. Here Δ is defined as the hyperspectral hue angle minus the multispectral hue angle. While the bands of MERIS in the blue are well-suited to retrieve the colour of oceanic waters with a hue angle above 170°, MERIS slightly underestimates the hue angle of green waters (hues between 70 to 170°) and overestimates the angle of CDOM rich waters (α < 70°). The drawn line is a polynomial approximation of this deviation Δ. If *a* = MERIS α/100, Δ can be approximated by:

Δ = −*12.05a^5^* + *88.93a^4^* − *244.70a^3^* + *305.241a^2^* − *164.70a* + *28.53* (15)

This seems to be a kind of overkill to retrieve the most accurate colour and indeed for this test data set and for the MERIS band settings the corrections are small. However, for other instruments with less favourable band settings we will demonstrate that Equation (15) becomes more important. This polynomial is, however, not to be used for angles below 37°, because the coefficients are based on a fit over the interval 37 to 230° and the 5th order polynomial results for Δ will give incorrect results outside this interval.

To summarize: to retrieve the true colour of open water from the MERIS instrument three steps are required; (1) calculate the (*X*, *Y*, *Z*) from the *R*_RS_ spectrum and the coefficients (Equation (14) with coefficients given in Table 2); (2) calculate the corresponding hue angle (Equations (10) and (11); and (3) add a small correction Δ (Equation (15)).

**Figure 6:**
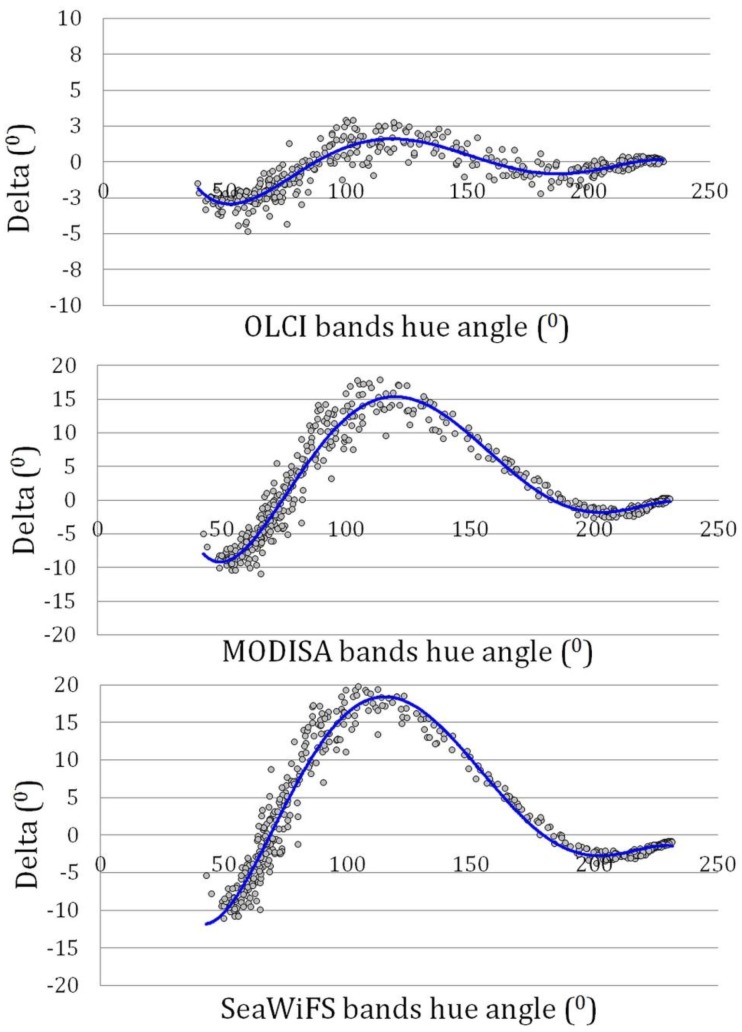
Deviation delta (°) from the hyperspectral hue angle as function of OLCI, MODIS and SeaWiFS hue angles derived from linear satellite band combinations. Note the change in the vertical scale from upper to lower panel.

The same calculations were done for the OLCI instrument on Sentinel-3, MODIS and SeaWiFS. The band coefficients are given in Table 3, the relation between the deviation delta (°) from the calculated hyperspectral hue angle as function of the derived hue from linear satellite band combinations is depicted in Figure 6. The polynomial coefficients are given in Table 4. All satellites show the same patterns, but the deviations become more severe if fewer bands are present. All four instruments estimate hue angle well for the blue waters, but have more problems for the green waters (mesotrophic to eutrophic algae dominated waters). The large deviation of MODIS and SeaWiFS in the green spectra is due to the missing band information at 620 nm. According to Table 2 and Table 3, a dominant contribution for red and green comes from the band at 620 nm on MERIS and OLCI, while for MODIS and SeaWiFS this information comes from the band near 550 nm (band 12 for MODIS and band 5 for SeaWiFS).

**Table 3:** Linear coefficients to calculate the chromaticity values based on OLCI (OL), MODIS (MO) and SeaWiFS (SW) bands.

**OL-Band**	**1**	**2**	**3**	**4**	**5**	**6**	**7**	**8**	**9**	**10**	**11**	
λ (nm)	400	413	443	490	510	560	620	665	673.5	681.25	708.75	710
xOL	0.154	2.957	10.861	3.744	3.750	34.687	41.853	7.323	0.591	0.549	0.189	0.006
yOL	0.004	0.112	1.711	5.672	23.263	48.791	23.949	2.836	0.216	0.199	0.068	0.002
zOL	0.731	14.354	58.356	28.227	4.022	0.618	0.026	0.000	0.000	0.000	0.000	0.000
**MO-Band**		**8**	**9**	**10**	**11**	**12**	**13**	**14**				
λ (nm)	400	412.5	443	490	531	551	667	678	710			
xMO	0.154	2.957	10.861	4.031	3.989	49.037	34.586	0.829	0.222			
yMO	0.004	0.112	1.711	11.106	22.579	51.477	19.452	0.301	0.080			
zMO	0.731	14.354	58.356	29.993	2.618	0.262	0.022	0.000	0.000			
**SW-Band**		**1**	**2**	**3**	**4**	**5**	**6**					
λ (nm)	400	413	443	490	510	555	670	710				
xSW	0.154	2.957	10.861	3.744	3.455	52.304	32.825	0.364				
ySW	0.004	0.112	1.711	5.672	21.929	59.454	17.810	0.132				
zSW	0.731	14.354	58.356	28.227	3.967	0.682	0.018	0.000				

**Table 4:** Polynomial coefficients to correct each instrument. a is the hue angle divided by 100.

Instrument	a^5^	a^4^	a^3^	a^2^	a^1^	Constant
MERIS	−12.0506	88.9325	−244.6960	305.2361	−164.6960	28.5255
OLCI	−12.5076	91.6345	−249.8480	308.6561	−165.4818	28.5608
MODISA	−48.0880	362.6179	−1011.7151	1262.0348	−666.5981	113.9215
SeaWiFS	−49.4377	363.2770	−978.1648	1154.6030	−552.2701	78.2940

### 3.2. Field Spectra

After we have derived these simple rules to calculate the colour coordinates from modelled multi-spectral information, we proof next that the number of bands and the position of these bands is sufficient to measure the intrinsic water colour accurately for *R*_RS_ spectra collected in the field. For 603 *R*_RS_ spectra from the central North Sea and Dutch coastal and inland waters, the hyperspectral hue angle was calculated. The multi-spectral satellite information was constructed, based on the extraction of the *R*_RS_ at the position of MERIS, MODIS, SeaWiFS and OLCI bands. Subsequently, the rules given above were applied for each instrument and the best approximation of the hue angle was derived.

**Figure 7:**
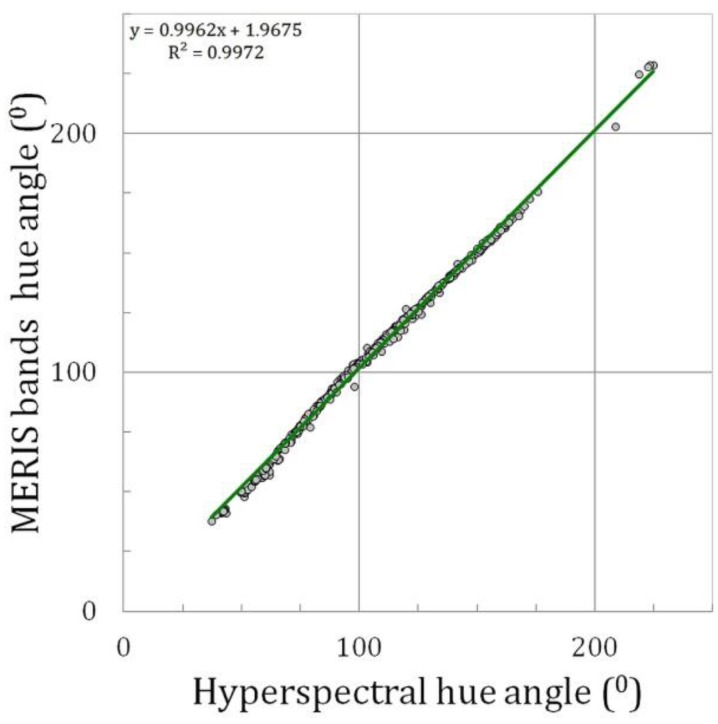
Example of the hue angle (“the colour”) reconstruction for MERIS. Hyperspectral hue angle (TriOS-Ramses) *versus* MERIS band hue angle after polynomial fit. The plot includes the linear fit line.

Figure 7 shows the correlation between the hyperspectral hue angle (TriOS-Ramses) and MERIS band hue angle after polynomial fit. It shows that for a large range in natural water colours, this algorithm is able to derive the true colour with very high accuracy. The slope is very close to 1.00 and the offset remains small.

The same results were derived for the MODIS band setting. Again, the slope is close to 1.00, although there is a slightly larger deviation, notably in the green region (70–130°). This shows that the final polynomial-based correction for the satellite derived colour depends still somewhat on the underlying spectral distribution. Nevertheless, the original correction derived from the set of 500 synthetic spectra still has the effect of bringing the data on the 1:1 line with respect to the true colour, as derived from the hyperspectral measurements.

### 3.3. Satellite Inter-Comparison

**Figure 8:**
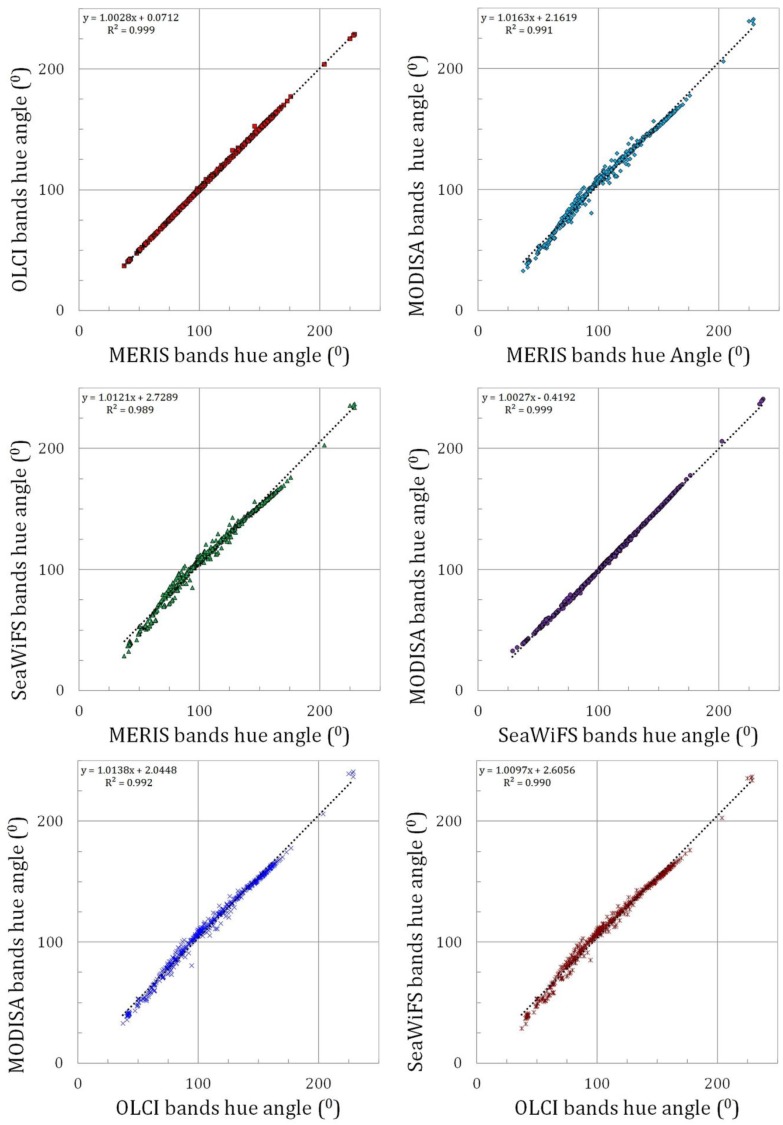
Inter-comparison of the hue angle (“the colour”) reconstruction of four ocean colour satellite sensors. Dotted lines give the best linear fit to the data.

As a test of the consistency between the algorithms for the four satellite band radiometers the derived hue angles were compared directly between pairs of instruments. The results were surprisingly good, as is shown in Figure 8 (slopes close to 1 with a *R*^2^ of 1). The imaging spectrometer band settings can be separated in two groups, (1) the MERIS and OLCI band setting that follow similar principles; and (2) the MODIS band setting that is partly an heritage of the SeaWiFS band setting. This is illustrated by the deviation angle at hue angle of 120° in Figure 5 and Figure 6 (typically 3° for MERIS and 15° for MODIS). However, after the polynomial correction this separation becomes less obvious. Although the pairs MODIS-SeaWiFS (Figure 8, middle pane right) and MERIS-OLCI (Figure 8, upper pane left) have the highest correlation, all relations do not show large systematic differences and have slopes close to 1.00.

**Figure 9:**
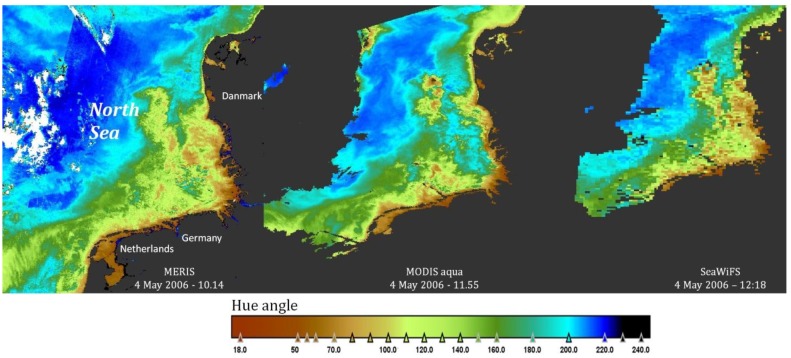
Hue angle image processing of MERIS, MODISA and SeaWiFS (left to right) with the code proposed in this manuscript. Area North Sea, date 4 May 2006.

This opens the way to use the hue angle as a parameter that compares very well between instruments. This is illustrated in Figure 9. A MERIS, MODIS and SeaWiFS image, all collected over the North Sea at 4 May 2006, were processed into the hue angle with the algorithm presented in this paper. The arithmetic code, to be used with the BEAM-VISAT software [21], can be downloaded as a Supplementary (see Supplementary) to this paper.

## 4. Discussion and Conclusions

In this paper we have demonstrated that for natural waters the complex spectral distribution of *R*_RS_ between 400 and 710 nm can be captured in one simple unit, the hue angle, by using the same methodology as in the science of chromaticity. The true colour angle can be approximated by a set of simple rules that consist of a weighted linear sum of the *R*_RS_ and a polynomial correction term that depends on the band setting of the imaging spectrometer.

The remote sensing reflection from natural waters show a broad variety in spectra, both in the magnitude and distribution of spectral features. However, the spectral distribution is not arbitrary; narrow spectral features are not very prominent and spectra change quite smoothly when the water composition changes [22]. The main reason for this is that spectral curves that describe the inherent optical properties of water are rather smooth. For example the absorption by CDOM and the scattering by silt and algae can be described by an exponential or power law type dependence on wavelength in the visual domain [17]. The absorption by phytoplankton is more complex with a strong absorption in the blue (400–500 nm), that can vary markedly as a function of the pigment composition, especially in coastal seas [23]. The red absorption band around 672 nm and the fluorescence by phytoplankton around 682 nm is also pronounced, but is only determined by the main pigment chlorophyll-a.

In the construction of ocean colour instruments all these aspects have been studied in detail and the band setting of these instruments has been carefully tuned to the expected colour variation [2,3]. A number of bands have been situated to capture the dynamics in the blue (413, 440, 490 and 510 nm) and the absorption/emission of the red band (672 and near 682 nm). It is this combination of careful band setting and smooth underlying variation in the Rrs that allows reliable retrieval of the colour hue angle.

What is the value of this hue angle and how can it be used in the future interpretation of sensor data for scientists who study the biology and hydrology of oceans, lakes and estuaries? It is important to realize that satellite *R*_RS_ derived from standard image processing is already corrected for atmospheric influences and illumination conditions and is therefore a quasi-inherent optical property. Also the calculated hue angle is a well-defined physical quantity that is linked directly to the composition and inherent optical properties of optically-deep water [10].

When the information of the individual bands is compressed in one variable, the hue angle, it is obvious that information is lost. However, the hue angle is not meant to replace existing (multi-spectral) information; it can merely be seen as a supplement to the existing water quality algorithms and wealth of other satellite products. For the open sea and ocean, the intrinsic colour is directly coupled to the variation in surface plankton concentration (case-1 waters [24]). In this specific case a direct relation between the hue angle and the chlorophyll-a can be established, as was shown by HydroLight [16] simulations in [12]. The next step would be to characterize the optically active constituents associated with each hue angle. Thus, the hue angle could be the entry into a look-up table to give properties such as chlorophyll concentration, CDOM and suspended sediments. Finally, the hue angle can also be transferred into a one-decimal Forel-Ule number [25] to compare small differences in the intrinsic colour and still be consistent with the long time series of a century-long monitoring of the Forel-Ule colour [26]. Even for the study of local processes, under for instance the influence of strong tides, the hue angle can reveal new insights.

The hue angle is a parameter that can be mapped over large areas and monitored in time for the operational time of all ocean colour imaging spectrometers. Of course, like all the other derived parameters, the final results are dependent on the calibration and atmospheric correction procedures of each instrument [27]. We have shown in this paper that the hue angles derived from different instruments can be compared in a simple way and therefore the method presented here can be used for vicarious calibration or matching of satellite and in-situ spectra (e.g., AERONET SEAPRISM [28]). It is a parameter that is calculated directly from the radiometric data and is not influenced by satellite-specific algorithms, like for example the chlorophyll-a concentration. This is well illustrated in Figure 9, in which actual satellite data are used. Given the nearly perfect agreement between MODIS and SeaWiFS in Figure 8, it is expected that the two images of those instruments are nearly identical. But they are not. Although it is always possible that the minor difference in overpass time (only 23 min) creates a different map due to mixing events (wind stress or tides), it might also point to inconsistencies in the calibration and atmospheric correction algorithms of the two instruments. In the future it would be interesting to investigate these differences in hue angle as a function of hue angle or composition to get more information on the origin of these differences. The presented algorithms bring the specific satellite product at a level for direct comparison. Therefore, we are confident that the methodology presented here offers a new way to compare observations and specific performance of imaging spectrometers over natural waters.

## Supplementary Files

Supplementary File 1
