# Supplementary material for: True Colour Classification of Natural Waters with Medium-Spectral Resolution Satellites: SeaWiFS, MODIS, MERIS and OLCI
Source: Sensors (Basel). 2015 Oct 9;15(10):25663–80. doi: 10.3390/s151025663 (PMC4634488; doi:10.3390/s151025663)
Supplement: Supplementary File 1 [file sensors-15-25663-s001.pdf]

OPEN ACCESS

sensors

ISSN 1424-8220
www.mdpi.com/journal/sensors

Supplementary Information

True Colour Classification of Natural Waters with Medium-Spectral Resolution Satellites: SeaWiFS, MODIS, MERIS and OLCI. *Sensors* 2015, *15*, 25663-25680

Hendrik J. van der Woerd [1,2,*] and Marcel R. Wernand [1,*]

1 Royal Netherlands Institute for Sea Research, Physical Oceanography, Marine Optics & Remote Sensing, PO box 59, Den Burg1790AB, Texel, The Netherlands
2 Institute for Environmental Studies (IVM), VU University Amsterdam, De Boelelaan 1087, Amsterdam 1081HV, The Netherlands

* Authors to whom correspondence should be addressed;
E-Mails: Hans.van.der.Woerd@nioz.nl (H.J.W.); Marcel.Wernand@nioz.nl (M.R.W.);
Tel.: +31-222-369-300 (M.R.W.); Fax: +31-222-369-417 (M.R.W.).

This document contains the band arithmetic to derive the hue angle and Forel-Ule number from multi-wavelength sensors MERIS, MODIS, SeaWiFS and OLCI.

The commands are adapted to the structure in the BEAM-VISAT software environment.

MERIS

X3
2.957*reflec_1 + 10.861*reflec_2 + 3.744*reflec_3 + 3.750*reflec_4 + 34.687*reflec_5 + 41.853*reflec_6 + 7.619*reflec_7 + 0.844*reflec_8 + 0.189*reflec_9

Y3
0.112*reflec_1 + 1.711*reflec_2 + 5.672*reflec_3 + 23.263*reflec_4 + 48.791*reflec_5 + 23.949*reflec_6 + 2.944*reflec_7 + 0.307*reflec_8 + 0.068*reflec_9

Z3
14.354*reflec_1 + 58.356*reflec_2 + 28.227*reflec_3 + 4.022*reflec_4 + 0.618*reflec_5 + 0.026*reflec_6 + 0.000*reflec_7 + 0.000*reflec_8 + 0.000*reflec_9

Chrx
X3/(X3 + Y3 + Z3)

Chry
Y3/(X3 + Y3 + Z3)
HUEMERIS
((atan2((Chry − 0.333333), (Chrx − 0.333333)))*180/ PI) < 0? ((atan2((Chry − 0.333333), (Chrx − 0.333333)))*180/PI) + 360:((atan2((Chry − 0.333333), (Chrx − 0.333333)))*180/PI)

HUEMERIS 100
HUEMERIS/100

POLYHueMERIS
−12.0506*pow(HUEMERIS 100,5) + 88.9325*pow(HUEMERIS 100,4) −244.6960*pow(HUEMERIS 100,3) + 305.2361*pow(HUEMERIS 100,2) − 164.6960* HUEMERIS 100 + 28.5255

HUEMERISPcorr
HUEnewMERIS + POLYHueMERIS

FUMERISPcorr
(HUEMERISPcorr > 232? 0:HUEMERISPcorr > 227.168? 1:(HUEMERISPcorr > 220.977? 2:(HUEMERISPcorr > 209.994? 3:(HUEMERISPcorr > 190.779? 4:(HUEMERISPcorr > 163.084? 5:(HUEMERISPcorr > 132.999? 6:(HUEMERISPcorr > 109.054? 7:(HUEMERISPcorr > 94.037? 8:(HUEMERISPcorr > 83.346? 9:(HUEMERISPcorr > 74.572? 10:(HUEMERISPcorr > 67.957? 11:(HUEMERISPcorr > 62.186? 12:(HUEMERISPcorr > 56.435? 13:(HUEMERISPcorr > 50.665? 14:(HUEMERISPcorr > 45.129? 15:(HUEMERISPcorr > 39.769? 16:(HUEMERISPcorr > 34.906? 17:(HUEMERISPcorr > 30.439? 18:(HUEMERISPcorr > 26.337? 19:(HUEMERISPcorr > 22.741? 20:(HUEMERISPcorr > 19? 21:(HUEMERISPcorr < 19? 21:0)))))))))))))))))))))))

OLCI

X3
0.154*reflec_1 + 2.957*reflec_2 + 10.861*reflec_3 + 3.744*reflec_4 + 3.750*reflec_5 + 34.687*reflec_6 + 41.853*reflec_7 + 7.323*reflec_8 + 0.591*reflec_9 + 0.549*reflec_10 + 0.189*reflec_11

Y3
0.004*reflec_1 + 0.112*reflec_2 + 1.711*reflec_3 + 5.672*reflec_4 + 23.263*reflec_5 + 48.791*reflec_6 + 23.949*reflec_7 + 2.836*reflec_8 + 0.216*reflec_9 + 0.199*reflec_10 + 0.068*reflec_11

Z3
0.731*reflec_1 + 14.354*reflec_2 + 58.356*reflec_3 + 28.227*reflec_4 + 4.022*reflec_5 + 0.618*reflec_6 + 0.026*reflec_7 + 0.000*reflec_8 + 0.000*reflec_9 + 0.000*reflec_10 + 0.000*reflec_11

Chrx
X3/(X3 + Y3 + Z3)

Chry
Y3/(X3 + Y3 + Z3)

HUESentinel3
((atan2((Chry − 0.333333), (Chrx − 0.333333)))*180/PI) < 0? ((atan2((Chry − 0.333333), (Chrx − 0.333333)))*180/PI) + 360:((atan2((Chry − 0.333333), (Chrx − 0.333333)))*180/PI)
HUESentinel3 100
HUESentinel3/100

POLYHueSentinel3
−12.5076*pow(HUESentinel3 100,5) + 91.6345*pow(HUESentinel3 100,4) −249.8480*pow(HUESentinel3 100,3) + 308.6561*pow(HUESentinel3 100,2) − 165.4818* HUESentinel3 100 + 28.5608

HUESentinel3Pcorr
HUEnewSentinel3 + POLYHueSentinel3

FUSentinel3Pcorr
(HUESentinel3Pcorr > 232? 0:HUESentinel3Pcorr > 227.168? 1:(HUESentinel3Pcorr > 220.977? 2:(HUESentinel3Pcorr > 209.994? 3:(HUESentinel3Pcorr > 190.779? 4:(HUESentinel3Pcorr > 163.084? 5:(HUESentinel3Pcorr > 132.999? 6:(HUESentinel3Pcorr > 109.054? 7:(HUESentinel3Pcorr > 94.037? 8:(HUESentinel3Pcorr > 83.346? 9:(HUESentinel3Pcorr > 74.572? 10:(HUESentinel3Pcorr > 67.957? 11:(HUESentinel3Pcorr > 62.186? 12:(HUESentinel3Pcorr > 56.435? 13:(HUESentinel3Pcorr > 50.665? 14:(HUESentinel3Pcorr > 45.129? 15:(HUESentinel3Pcorr > 39.769? 16:(HUESentinel3Pcorr > 34.906? 17:(HUESentinel3Pcorr > 30.439? 18:(HUESentinel3Pcorr > 26.337? 19:(HUESentinel3Pcorr > 22.741? 20:(HUESentinel3Pcorr > 19? 21:(HUESentinel3Pcorr < 19? 21:0)))))))))))))))))))))

MODIS

X3
2.957*Rrs_412 + 10.861*Rrs_443 + 4.031*Rrs_488 + 3.989*Rrs_531 + 49.037*Rrs_555 + 34.586*Rrs_667 + 0.829*Rrs_678

Y3
0.112*Rrs_412 + 1.711*Rrs_443 + 11.106*Rrs_488 + 22.579*Rrs_531 + 51.477*Rrs_555 + 19.452*Rrs_667 + 0.301*Rrs_678

Z3
14.354*Rrs_412 + 58.356*Rrs_443 + 29.993*Rrs_488 + 2.618*Rrs_531 + 0.262*Rrs_555 + 0.000*Rrs_667 + 0.000*Rrs_678

Chrx
X3/(X3 + Y3 + Z3)

Chry
Y3/(X3 + Y3 + Z3)

HUEMODISa
((atan2((Chry − 0.333333), (Chrx − 0.333333)))*180/PI) < 0? ((atan2((Chry − 0.333333), (Chrx − 0.333333)))*180/PI) + 360:((atan2((Chry − 0.333333), (Chrx − 0.333333)))*180/PI)

HUEMODISa 100
HUEMODISa/100

POLYHueMODISa
−48.0880*pow(HUEMODISa 100,5) + 362.6179*pow(HUEMODISa 100,4) − 1011.7151*pow(HUEMODISa 100,3) + 1262.0348*pow(HUEMODISa 100,2) − 666.5981* HUEMODISa 100 + 113.9215

HUEMODISaPcorr
HUEnewMODISa + POLYHueMODISa

FUMODISaPcorr
(HUEMODISaPcorr > 232? 0:HUEMODISaPcorr > 227.168? 1:(HUEMODISaPcorr > 220.977? 2:(HUEMODISaPcorr > 209.994? 3:(HUEMODISaPcorr > 190.779? 4:(HUEMODISaPcorr > 163.084? 5:(HUEMODISaPcorr > 132.999? 6:(HUEMODISaPcorr > 109.054? 7:(HUEMODISaPcorr > 94.037? 8:(HUEMODISaPcorr > 83.346? 9:(HUEMODISaPcorr > 74.572? 10:(HUEMODISaPcorr > 67.957? 11:(HUEMODISaPcorr > 62.186? 12:(HUEMODISaPcorr > 56.435? 13:(HUEMODISaPcorr > 50.665? 14:(HUEMODISaPcorr > 45.129? 15:(HUEMODISaPcorr > 39.769? 16:(HUEMODISaPcorr > 34.906? 17:(HUEMODISaPcorr > 30.439? 18:(HUEMODISaPcorr > 26.337? 19:(HUEMODISaPcorr > 22.741? 20:(HUEMODISaPcorr > 19? 21:(HUEMODISaPcorr < 19? 21:0))))))))))))))))))))))

SeaWiFS

X3
2.957*Rrs_412 + 10.861*Rrs_443 + 3.744*Rrs_490 + 3.455*Rrs_510 + 52.304*Rrs_555 + 32.825*Rrs_670

Y3
0.112*Rrs_412 + 1.711*Rrs_443 + 5.672*Rrs_490 + 21.929*Rrs_510 + 59.454*Rrs_555 + 17.810*Rrs_670

Z3
14.354*Rrs_412 + 58.356*Rrs_443 + 28.227*Rrs_490 + 3.967*Rrs_510 + 0.682*Rrs_555 + 0.018*Rrs_670

Chrx
X3/(X3 + Y3 + Z3)

Chry
Y3/(X3 + Y3 + Z3)

HUESeaWiFS
((atan2((Chry − 0.333333), (Chrx − 0.333333)))*180/PI)< 0? ((atan2((Chry − 0.333333), (Chrx − 0.333333)))*180/PI)+360:((atan2((Chry − 0.333333), (Chrx − 0.333333)))*180/PI)

HUESeaWiFS 100
HUESeaWiFS/100

POLYHueSeaWiFS
−49.4377*pow(HUESeaWiFS 100,5) + 363.2770*pow(HUESeaWiFS 100,4) − 978.1648*pow(HUESeaWiFS 100,3) + 1154.6030*pow(HUESeaWiFS 100,2) − 552.2701*HUESeaWiFS 100 + 78.2940

HUESeaWiFSPcorr
HUEnewSeaWiFS + POLYHueSeaWiFS

FUSeaWiFSPCorr
(HUESeaWiFSPcorr > 232? 0:HUESeaWiFSPcorr > 227.168? 1:(HUESeaWiFSPcorr > 220.977? 2:(HUESeaWiFSPcorr > 209.994? 3:(HUESeaWiFSPcorr > 190.779? 4:(HUESeaWiFSPcorr >

163.084? 5:(HUESeaWiFSPcorr > 132.999? 6:(HUESeaWiFSPcorr > 109.054? 7:(HUESeaWiFSPcorr > 94.037? 8:(HUESeaWiFSPcorr > 83.346? 9:(HUESeaWiFSPcorr > 74.572? 10:(HUESeaWiFSPcorr > 67.957? 11:(HUESeaWiFSPcorr > 62.186? 12:(HUESeaWiFSPcorr > 56.435? 13:(HUESeaWiFSPcorr > 50.665? 14:(HUESeaWiFSPcorr > 45.129? 15:(HUESeaWiFSPcorr > 39.769? 16:(HUESeaWiFSPcorr > 34.906? 17:(HUESeaWiFSPcorr > 30.439? 18:(HUESeaWiFSPcorr > 26.337? 19:(HUESeaWiFSPcorr > 22.741? 20:(HUESeaWiFSPcorr > 19? 21:(HUESeaWiFSPcorr < 19? 21:0))))))))))))))))))))))

© 2015 by the authors; licensee MDPI, Basel, Switzerland. This article is an open access article distributed under the terms and conditions of the Creative Commons Attribution license (http://creativecommons.org/licenses/by/4.0/).
